# 

**Published:** 1897-06-26

**Authors:** 


### EARLY EDITION.

**Notices of Births, Deaths, and Marriages** are inserted in this column at a charge of 2s. 6d. for an announcement not exceeding four lines.

### NOTICE TO CORRESPONDENTS.

All MS., Letters, Books for Review, and other matters intended for the Editor should be addressed The Editor, "The Hospital," The Hospital Building, 28 & 29, Southampton Street, Strand, W.C.

The Editor cannot undertake to return rejected MS., even when accompanied by stamped directed envelope.

**Notice to Advertisers and Subscribers.**

All Advertisements, Orders for Copies of The Hospital, and Business Communications should be addressed to **THE MANAGER, (Not to The Editor),** The Hospital, The Hospital Building, 28 & 29, Southampton Street, Strand, London, W.C.

The Cost of Subscription, post free, is as follows:—Per week, 2½d.; per quarter, 2s. 8d.; per half-year, 5s. 3d.; per annum, 10s. 6d. Foreign:—Per annum, 15s. 2d.; payable, in all cases, in advance.

### SCRAPS AND GLEANINGS.

The Royal Hospital for Diseases of the Chest, City Road, has received a donation of £100 from the trustees of Smith's Charity.

Baroness Hirsch has given £100 towards the extension fund for the City Orthopædic Hospital, Hatton Garden.

During 1896 the sum of £609 1s. was subscribed by the employés of J. S. Fry and Sons (Limited) and distributed among the following medical and other institutions; Bristol Dispensary (for notes), £210; General Hospital, £140; Royal Infirmary, £140; Children's Hospital, £55; Hospital for Skin Diseases (for notes), £27 6s.; Homœopathic Dispensary, £10 10s.; Weston Sanatorium, £10 10s.; Eye Hospital, Maudlin Street, £10 10s.; Home of Rest, Durdham Down, £2 2s.; Torquay Hospital, £2 2s.; Convalescent Home, Walton, Clevendon, £1 1s. In addition to the above, the mechanics and those engaged in the building department subscribed £29, which has been distributed amongst the various medical institutions.

